# Resistance saturation in semi-conducting polyacetylene molecular wires

**DOI:** 10.1007/s10825-023-02043-7

**Published:** 2023-05-15

**Authors:** Angelo Valli, Jan M. Tomczak

**Affiliations:** 1 Department of Theoretical Physics, Institute of Physics, Budapest University of Technology and Economics, Műegyetem rkp. 3., Budapest, H-1111 Hungary; 2 Institute for Theoretical Physics, Vienna University of Technology, Wiedner Hauptstrasse 8-10, A-1040 Vienna, Austria; 3 Department of Physics, King’s College London, Strand, London, WC2R 2LS UK; 4 Institute for Solid State Physics, Vienna University of Technology, Wiedner Hauptstrasse 8-10, A-1040 Vienna, Austria

**Keywords:** Mesoscopic systems, Transport properties, Landauer and Kubo approach, Electronic correlations

## Abstract

Realizing the promises of molecular electronic devices requires an understanding of transport on the nanoscale. Here, we consider a Su-Schrieffer-Heeger model for semi-conducting trans-polyacetylene molecular wires in which we endow charge carriers with a finite lifetime. The aim of this exercise is two-fold: (i) the simplicity of the model allows an insightful numerical and analytical comparison of the Landauer and Kubo linear-response formalism; (ii) we distill the prototypical characteristics of charge transport through gapped mesoscopic systems and compare these to bulk semiconductors. We find that both techniques yield a residual differential conductance at low temperatures for contacted polyacetylene chains of arbitrary length—in line with the resistivity saturation in some correlated narrow-gap semiconductors. Quantitative agreement, however, is limited to not too long molecules. Indeed, while the Landauer transmission is suppressed exponentially with the system size, the Kubo response only decays hyperbolically. Our findings inform the choice of transport methodologies for the ab initio modelling of molecular devices.

## Introduction

The last decades have witnessed tremendous experimental progress in the field of nanoelectronics, pushing investigation of transport properties toward the ultimate scale of single-molecule architectures [1–4]. At the nanoscale, semi-classical approaches break down as the nature of electron transport is inherently quantum mechanical [5, 6], showing phenomena ranging from conductance quantization [7] and quantum interference [2, 8], to the anomalous quantum Hall effect [9]. At the length scale of individual molecules in, e.g., a mechanical break-junction setup [2–4], electron transport is essentially ballistic. However, in complex quantum devices, both the coupling to the environment and to internal degrees of freedom of the molecular bridge entail sources of incoherence for the charge carriers. A fundamental theoretical understanding of the mechanisms of quantum transport and the role of incoherence effects across multiple length scales is challenging [4, 10]. At the same time, it is of pivotal importance to the interpretation of experiments [3] and to eventually harvest the potential of quantum technologies for next-generation nanoelectronic devices.

Among the most prominent methodologies to describe transport phenomena on the nanoscale are the Landauer-Büttiker formalism and Kubo linear response [11–14]. In fact, it has been established that the Landauer formula [12] itself can be derived from linear response [15–17]. It was motivated that the omission of higher order terms (vertex corrections) in the Landauer transmission is admissible even for interacting electrons when, at zero temperature, inelastic processes subside [18]. In general, however, the current has to be recast in terms of an effective transmission kernel, where the Green’s function is dressed by a many-body self-energy and the molecule-lead coupling is renormalized by vertex corrections [19–22]. While in practice evaluating such vertex corrections is challenging and thus not often attempted, numerical results seem to suggest [19] that off-resonant, near-equilibrium transport can still be qualitatively described in terms of *quasi*-particle scattering.

Using the Kubo formula, it was recently shown [23, 24] that finite lifetimes of charge carriers can lead to non-trivial transport phenomena in *bulk* correlated narrow-gap semiconductors [25]. From this perspective, it is interesting to ask how quasi-particle incoherence manifests in transport through semiconducting *nanoscopic* systems. The similarities of the Landauer and the Kubo formalism for coherent transport further raise the question how differently the charge carriers’ finite lifetimes are reflected in the two approaches.

To answer these questions, we investigate in this paper the transport through a simple semiconducting molecule, *trans*-polyacetylene. For the charge carriers, we impose an *ad hoc* finite lifetime that serves to mimic scattering events taking place within the molecule, e.g., from electron–electron scattering, disorder or electron–phonon coupling. We find quasi-particle incoherence to lead to significant, qualitative changes in the molecule’s conductive properties. In particular, we find the resistance to saturate below a surprisingly large characteristic temperature. Further, we reveal marked differences between the Kubo and the Landauer results. In fact, we motivate—numerically and analytically—that the Kubo formalism is more general, in the sense that it contains the Landauer conductance as its small-scattering rate limit. Our results advocate that future investigations of semiconducting organic systems—and devices build from them—need to be mindful of the influence of incoherence onto transport properties—even at room temperature.

## System and model

Polyacetylene (PA) is an organic conjugated polymer with chemical formula (CH)$_n$. Of particular interest is the structure of *trans*-polyacetylene which dimerizes due to electron–phonon coupling through a Peierls mechanism. Thus, trans-PA consists of alternating single ($\mathrm {C\!-\!C}$) and double ($\mathrm {C\!=\!C}$) carbon bonds. Experimental investigations suggest bond-lengths $d_{\mathrm {C-C}}={1.44}$Å and $d_{\mathrm {C=C}}={1.36}$Å [26, 27], with a relative angle $\gamma \approx$ 122$^\circ$, yielding a unit cell length of $d=2a=a_1+a_2 \approx$ 2.45Å, where *a* is the lattice spacing of the undimerized chain, whereas $a_1$ and $a_2$ are the projection of the single- and double-bonds along the chain, respectively. Following the seminal work of Su, Schrieffer, and Heeger [28], a single dimerized PA chain with *N* unit cells can be described within an effective tight-binding model for $\pi$-electrons with nearest-neighbor hopping

1 $$\begin{aligned} H_{\textrm{SSH}} = \sum _{i=1}^{2N-1}\sum _{\sigma } (t_0 - 2\alpha u_i) c^{\dagger }_{i+1,\sigma }c^{}_{i\sigma } + \mathrm {h.c.}, \end{aligned}$$

where $c^{(\dagger )}_{i\sigma }$ is the annihilation (creation) operator for an electron on a C-p$_z$ atomic orbital (AO) *i* of spin $\sigma$, while $t_0={2.5}\textrm{eV}$ is the hopping integral for the undimerized chain, $\alpha ={4.1}{\text {eV}}/$Å is the electron–phonon coupling constant, and $u_i=(-1)^i u_0$ is the displacement describing the dimerization, with $u_0={0.04}$Å. With these parameters, the dimerized hoppings are given by $t_1=t_{\mathrm {C\!-\!C}}={2.172}\textrm{eV}$ and $t_2=t_{\mathrm {C=C}}={2.828}\textrm{eV}$, which yields a Peierls gap $\Delta _{\infty }=2(t_2-t_1)=8\alpha u_0={1.312}\textrm{eV}$ [28].

**Fig. 1:**
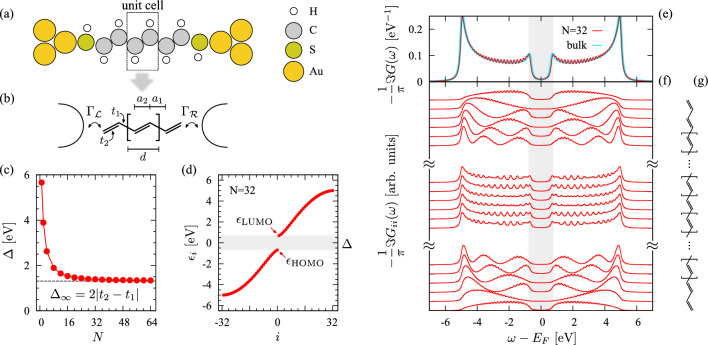
**a** Representations of a two-terminal junction, with a PA chain bridging Au electrodes through thiol (SH) anchoring groups. **b** Mapping to an SSH model, single (C−C) and double (C$=$C) bonds correspond to lattice spacing $a_1$ and $a_2$, respectively (with $d=a_1+a_2$ the length of the unit cell) while $\Gamma _\mathcal{L}$ and $\Gamma _\mathcal{R}$ describe the left and right molecule-lead coupling, respectively. **c** Evolution of the HOMO-LUMO gap as a function of the PA length *N* (unit cells) converging to the bulk Peierls gap $\Delta _{\infty }$ (dashed line). **d** Distribution of the MO eigenenergies $\epsilon _i$ for a $N=32$ PA chain. **e** Spectral function of bulk PA and a $N=32$ PA chain. **f** Site-resolved spectral function for selected C atoms *i* across the PA chain, at the edges and in the middle, as shown in (**g**). The grey area in (**d**, **e**, **f**) highlights the spectral gap $\Delta$. For clarity, the spectra in (**e**, **f**) are obtained with a broadening $\eta =0.1$ eV

The unit cell and the bond structure of PA, including the lattice spacing and the hopping, are shown in Fig. 1a, b. The Peierls gap between the highest occupied (HOMO) and the lowest unoccupied (LUMO) molecular orbitals is found to decay as 1/*N* towards the bulk Peierls gap $\Delta _{\infty }$, see Fig. 1c. The representative distribution of molecular orbital (MO) eigenenergies are shown in Fig. 1d for a $N=32$ PA chain. The spectral function of this PA chain, shown Fig. 1e, closely resembles the one of the infinitely-long PA chain (periodic bulk). For the components projected onto individual C’s AO in Fig. 1f, those towards the centre of the chain indeed resemble the spectral function of an infinitely long (dimerized) one-dimensional chain, delimited by its characteristic van Hove singularities. The spectrum close to the edges of the chain, however, assumes a shape that is semi-circular outside the hybridization gap. Analytical arguments (for the undimerized chain) supporting the numerical spectra are discussed in Appendix 1. All projected spectral functions are even, i.e., $\Im G_{ii}(\omega )=\Im G_{ii}(-\omega )$, which is a manifestation of the particle-hole symmetry of the model. Moreover, since the lattice is bipartite, the SSH Hamiltonian is invariant under a sublattice (chiral) symmetry which maps AOs $i \rightarrow 2N+1-i$.

The SSH model also raised interest in the community for its topological properties [29–31]. Here, we are taking the *intra*-cell and *inter*-cell hopping to be $t_2$ and $t_1$, respectively, with $t_2>t_1$. With this choice, the system is topologically trivial. However, PA can dimerize in another pattern, in which single- and double-bonds are interchanged, leading to a nonzero winding number and a topologically nontrivial state. Then, the SSH model supports zero-energy edge states and can host domain walls, giving rise to solitons [29, 30]. As a consequence, for varying termination, the PA chain displays dramatically different transport properties [32, 33], a complexity beyond the scope of this work.

## Electron transport theory

In a typical transport (two-terminal) setup, the system is divided into three regions, in which a central (or scattering) region (${\mathcal C}$) bridges left ($\mathcal{L}$) and right ($\mathcal{R}$) electrodes. The electrodes are connected at infinity to particle reservoirs which account for the dissipation necessary to establish a steady state. Each reservoir injects thermalized carriers which are distributed according to the Fermi-Dirac statistics at the equilibrium chemical potential $\mu _\mathcal{L}$ and $\mu _\mathcal{R}$, corresponding to a bias voltage $V_b = (\mu _\mathcal{L} - \mu _\mathcal{R})/e$, whereas the transport in the central region is phase-coherent. Then, the Hamiltonian has the following form

2 $$\begin{aligned} {\textbf{H}} = \begin{pmatrix} {\textbf{H}}_\mathcal{L} &{} {\textbf{H}}_{\mathcal{L}\mathcal{C}} &{} {\textbf{H}}_{\mathcal{L}\mathcal{R}} \\ {\textbf{H}}_{\mathcal{C}\mathcal{L}} &{} {\textbf{H}}_\mathcal{C} &{} {\textbf{H}}_{\mathcal{C}\mathcal{R}} \\ {\textbf{H}}_{\mathcal{R}\mathcal{L}} &{} {\textbf{H}}_{\mathcal{R}\mathcal{C}} &{} {\textbf{H}}_{\mathcal{R}} \end{pmatrix}, \end{aligned}$$

where ${\textbf{H}}_\mathcal{C}$ describes the scattering region, ${\textbf{H}}_\mathcal{L}$ and ${\textbf{H}}_\mathcal{R}$ describe the $\mathcal{L}$ and $\mathcal{R}$ lead, respectively, and the off-diagonal elements account for the coupling between these regions. Typically, it is required that the two leads are coupled only through the scattering region, i.e., ${\textbf{H}}_{\mathcal{L}\mathcal{R}}={\textbf{0}}$.^1^

Since the leads are infinitely extended, a Hamiltonian formulation of the scattering problem is not numerically feasible. Hence the leads are instead accounted for by embedding them into the scattering region. The retarded Green’s function of the scattering region then reads

3 $$\begin{aligned} {\textbf{G}}^{-1}(\omega ) = (\omega + \imath \eta ) {\textbf{I}}_\mathcal{C} - {\textbf{H}}_\mathcal{C} - {\mathbf \Sigma }_\mathcal{L}(\omega ) - {\mathbf \Sigma }_\mathcal{R}(\omega ) - {\mathbf \Sigma }_\mathcal{C}(\omega ) \end{aligned}$$

where the embedding self-energy of lead $\alpha$ is defined as

4 $$\begin{aligned} {\mathbf \Sigma }_{\alpha }(\omega ) = {\textbf{H}}_{\mathcal{C}\alpha } {\textbf{g}}_{\alpha }(\omega ) {\textbf{H}}_{\alpha \mathcal{C}}, \end{aligned}$$

in terms of the corresponding surface Green’s function

5 $$\begin{aligned} {\textbf{g}}^{-1}_{\alpha }(\omega ) = (\omega + \imath \eta + \mu _{\alpha }) {\textbf{I}}_{\alpha } - {\textbf{H}}_{\alpha }. \end{aligned}$$

There are different possible routes to take into account the electrodes [34, 35], and also the AO beyond the ones of the $\pi$-system [36]. For the sake of simplicity, in the following, we assume that the molecule-lead coupling is restricted to the AO at the edges of the PA chain ($\ell$ and *r*, respectively), and we employ a wide-band approximation [35] for the leads. Then, the embedding self-energy of each lead reduces to an imaginary constant, i.e., $({\mathbf \Sigma }_\mathcal{L})_{\ell \ell }=-\imath \Gamma _\mathcal{L}/2$ and $({\mathbf \Sigma }_\mathcal{R})_{rr}=-\imath \Gamma _\mathcal{R}/2$.

Finally, the self-energy ${\mathbf \Sigma }_\mathcal{C}$ describes the effects of electron–electron correlations within the scattering region. In the following, we employ a *phenomenological* description where the electronic correlations are limited to a static, constant and uniform scattering rate $\Gamma$ for every AO, i.e.,

6 $$\begin{aligned} {\mathbf \Sigma }_\mathcal{C}(\omega ) = -\imath \Gamma \ {\textbf{I}}_\mathcal{C}, \end{aligned}$$

implying that charge carriers have a finite lifetime $\tau =\hbar /(2\Gamma )$. In this work, we do not specify the microscopic origin of the scattering, which could be owing to, e.g., disorder, electron–phonon coupling or electronic correlations. More sophisticated approximations further include dynamical renormalizations, either limited to low-energies via the quasi-particle weight [37], or account for the full frequency dependence, e.g., within *GW*, [38–40] Anderson impurity calculations [41–44], dynamical mean-field theory [45, 46] or its extensions [47–50]. Such approaches can describe correlation phenomena at all energy scales, including site-selective Mott physics [51–54] and temperature-dependent phenomena [23, 24, 55–59], and have been successfully applied to describe the electronic [47, 53, 57, 60–64] and transport [21, 22, 38–40, 51, 52, 54–56, 58, 65–70] properties of molecular and nanoscopic systems.

In the following, we recap the Landauer and Kubo theory for electron transport within the Green’s function formalism. Making the formulae explicit for the SSH model will facilitate to highlight similarities and differences between the approaches.

### Landauer

Within the Landauer formalism, the electron transmission function through the scattering region is given by

7 $$\begin{aligned} T(\omega ) = \textrm{Tr} \Big [ \mathbf{\Gamma }_\mathcal{L}(\omega ) \textbf{G}^{\dagger }(\omega ) \mathbf{\Gamma }_\mathcal{R}(\omega ) \textbf{G}(\omega ) \Big ], \end{aligned}$$

where

8 $$\begin{aligned} \mathbf{\Gamma }_{\alpha }(\omega ) = \imath \Big [ \mathbf{\Sigma }^{}_{\alpha }(\omega ) - \mathbf{\Sigma }^{\dagger }_{\alpha }(\omega ) \Big ] \end{aligned}$$

encloses the spectral information of lead $\alpha$. For a deeper understanding of the channel structure of the electron transport, it is useful to make the trace in the Landauer formula (7) explicit, yielding

9 $$\begin{aligned} T(\omega ) = \sum _{ii'}\sum _{jj'} ({\mathbf \Gamma }_\mathcal{L})_{ii'} {\textbf{G}}^{\dagger }_{i'j}(\omega ) ({\mathbf \Gamma }_\mathcal{R})_{jj'} {\textbf{G}}_{j'i}(\omega ). \end{aligned}$$

Considering the approximation on the structure of the leads from above, $(\Gamma _\mathcal{L})_{ii'}\propto \delta _{i\ell }\delta _{i'\ell }$ and $(\Gamma _\mathcal{R})_{jj'}\propto \delta _{jr}\delta _{j'r}$, this reduces to

10 $$\begin{aligned} T(\omega ) = \Gamma _\mathcal{L}\Gamma _\mathcal{R} \vert \textbf{G}_{\ell r}(\omega )\vert ^2. \end{aligned}$$

Hence, the Landauer transmission is controlled by the (nonlocal) Green’s function that links the $\ell$ and *r* AOs, corresponding to the outer C atoms which are connected to the $\mathcal{L}$ and $\mathcal{R}$ lead, respectively.

Although a derivation from linear response is possible [15–17, 19], the Landauer transmission, Eq. (10), is *de facto* agnostic to the applied perturbation as it includes no information on external couplings. Indeed, Landauer describes the amplitude (absolute square) for the process of adding an electron on one end of the molecule (AO *r*) and removing it from the other (AO $\ell$),

11 $$\begin{aligned} \textbf{G}_{\ell r}(t-t^\prime )=-i\theta (t-t^\prime )\left\langle \{c^{}_{\ell \sigma }(t),c^\dagger _{r\sigma }(t^\prime ) \} \right\rangle . \end{aligned}$$

In this tunneling process (which is independent of spin σ), inequivalent bonds or effects of dimerization only manifest implicitly, via their effect onto the hopping amplitudes in the Hamiltonian.

Finally, we recall that, in the presence of many-body correlations within the scattering region, the Landauer transmission, Eq. (7) is approximate. Besides considering the many-body Green’s function, one also needs to include a vertex correction [19, 22, 41]. Following Ferretti et al. [41], the corrected expression reads

12 $$\begin{aligned} T(\omega ) = \textrm{Tr} \Big [ \mathbf{\Gamma }_\mathcal{L}(\omega ) \textbf{G}^{\dagger }(\omega ) \mathbf{\Gamma }_\mathcal{R}(\omega ) \mathbf{\Lambda }(\omega ) \textbf{G}(\omega ) \Big ], \end{aligned}$$

where

13 $$\begin{aligned} \mathbf{\Lambda }(\omega ) = \textbf{1} + \left( \mathbf{\Gamma }_\mathcal{L} + \mathbf{\Gamma }_\mathcal{R}\right) ^{-1} \mathbf{\Gamma }_\mathcal{C}, \end{aligned}$$

and $\mathbf{\Gamma }_\mathcal{C}=-2\Im \mathbf{\Sigma }_\mathcal{C}$ is defined in terms of the many-body self-energy of the scattering region. Considering the matrix structure of the leads’ and many-body self-energies, it is easy to verify that, including the vertex corrections, Eq. (10) becomes

14 $$\begin{aligned} T(\omega ) = \Gamma _\mathcal{L}(\Gamma _\mathcal{R}+{\Gamma }) \vert \textbf{G}_{\ell r}(\omega )\vert ^2. \end{aligned}$$

For all interpretational purposes, the vertex corrections correspond to an asymmetric renormalization of the molecule-lead coupling, introducing an effective *many-body electrode* in which the electrons undergo some scattering processes, before being re-injected into the system [22].

### Kubo

Within the Kubo formalism, the formula for the optical transmission along the *x* direction (when neglecting vertex corrections [71–75]) is given by [76]

15 $$\begin{aligned} T(\omega ) = \frac{2}{L^2} \textrm{Tr} \Big [ \hbar \textbf{v}_x \Im \textbf{G}(\omega ) \hbar \textbf{v}_x \Im \textbf{G}(\omega ) \Big ], \end{aligned}$$

where $L=Nd-a_1$ is the length of a PA chain containing *N* unit cells,^2^ the Green’s function including the embedding self-energy of the leads is given by Eq. (3) and the matrix elements of the velocity are given by

16 $$\begin{aligned} (\textbf{v}_x)_{ij} = \frac{\imath }{\hbar } {\textbf{t}}_{ij}(x_i-x_j). \end{aligned}$$

For lattice models, such as the SSH Hamiltonian Eq. (1), these velocities are typically derived from the Peierls substitution approach [78–80], in which the vector potential couples to the atomic positions (projected onto the direction of transport).

Making the trace explicit as above, and expressing the velocity in terms of the hopping, yields

17 $$\begin{aligned} T(\omega ) = -\frac{2}{L^2} \sum _{ii'} \sum _{jj'} {\textbf{t}}_{ii'}(x_{i'}-x_{i}) \Im {\textbf{G}}_{i'j}(\omega ) {\textbf{t}}_{jj'}(x_{j'}-x_{j}) \Im {\textbf{G}}_{j'i}(\omega ). \end{aligned}$$

For dimerized PA chains within the SSH model we get

18 $$\begin{aligned} T(\omega ) = -\frac{2}{L^2} \sum _{i,j=2}^{2N-1} {\textbf{t}}_{i,i\pm 1}(x_{i\pm 1}-x_{i})\Im {\textbf{G}}_{i\pm 1,j}(\omega ) {\textbf{t}}_{j,j\pm 1}(x_{j\pm 1}-x_{j}) \Im {\textbf{G}}_{j\pm 1,i}(\omega ). \end{aligned}$$

Contrary to Landauer, the Kubo approach, more manifestly, describes the response to a specific external perturbation. Further, the current vertices, or velocities, Eq. (16), have direct knowledge of the internal structure of the molecule, e.g., the dimerization and the buckling angle $\gamma$, as the electromagnetic vector potential couples to atomic positions.

**Fig. 2:**
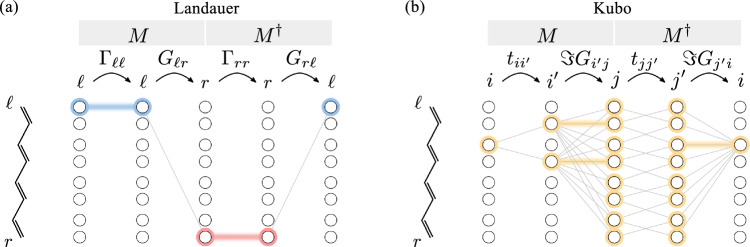
Schematic representation of the electron transport across the PA wire within the (**a**) Landauer and (**b**) Kubo formalism. The transmission amplitude, $\textrm{Tr}(MM^\dagger )$, consists of processes *M* and $M^{\dagger }$ that can be associated with paths in real-space (vertical axis). The Landauer transmission is given by a single path (i.e., from $\ell$ to *r*). The Kubo transmission is made up of many individual connections, that are built from nearest-neighbor hopping $t_{i,i\pm 1}$ and spectral functions $\Im G_{ij}$. The trace constrains each path to start and end at a given site *i*. In both panels, colorful circles highlight the sites involved at each step, while colorful horizontal lines highlight *local* processes

### Green’s functions and transmission paths in polyacetylene

Processes that contribute to the amplitude of the Landauer and the Kubo transmission are illustrated in Fig. 2a and b, respectively: The Landauer transmission, Eq. (9), is completely determined by the nonlocal Green’s function between the AO connected to the $\mathcal{L}$ and $\mathcal{R}$ reservoirs. The Kubo response, instead, consists of many contributions. Each contribution is built from nearest-neighbor hopping processes $t_{i,i\pm 1}$ and spectral functions $\Im \textbf{G}_{ij}$. In particular, some contributions involve *local* spectral functions $\Im \textbf{G}_{ii}$ (i.e., projected on a single AO *i*) which are highlighted by colorful lines in Fig. 2b.

**Fig. 3:**
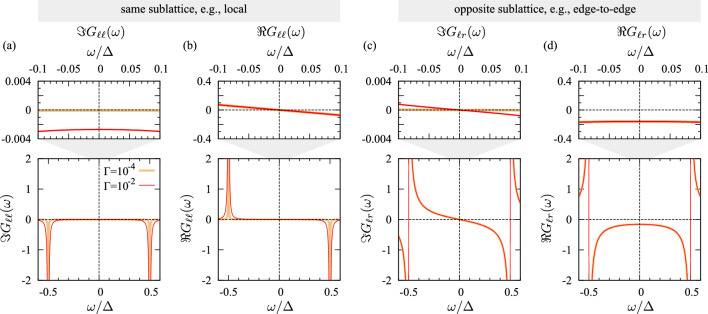
Representative Green’s function connecting (**a**, **b**) the same sublattice, $\textbf{G}_{\ell \ell }$, and (**c**, **d**) the opposite sublattice, $\textbf{G}_{\ell r}$, of C atoms in the PA chain. Sublattice one (two) consists of the C atoms with a double bond to their right (left). Clearly visible is that, at the Fermi level ($E_F=0$), the dominant spectral ingredient to the Kubo and the Landauer transmission are the even components $\Im \textbf{G}_{\ell \ell }$ and $\Re \textbf{G}_{\ell r}$, respectively. Noteworthy, only the former is strongly dependent on the scattering rate $\Gamma$, heralding differences between the Kubo and the Landauer transmission

Owing to the particle-hole symmetry of the SSH Hamiltonian, the Fermi energy falls in the middle of the HOMO-LUMO gap (and hence will be set to $E_F=0$ from here on) and the Green’s function components are either even or odd functions of frequency, see Fig. 3. Specifically, the following relations hold [66]

19 $$\begin{aligned} - \textbf{G}^{*}_{i,i+2n}(-\omega )&= \textbf{G}_{i,i+2n}(\omega ), \end{aligned}$$

20 $$\begin{aligned} \textbf{G}^{*}_{i,i+2n+1}(-\omega )&= \textbf{G}_{i,i+2n+1}(\omega ). \end{aligned}$$

This in particular implies $\Im \textbf{G}_{1,i+2n+1}(0)=0$, so that for all index pairs (*i*, *j*) with opposite parity (i.e., belonging to different sublattices) the corresponding contributions to the Kubo response vanish. Interestingly, at the Fermi energy, the contribution of $\Im \textbf{G}_{\ell r}$ is *absent* in the Kubo response. As is clearly visible in Fig. 3, the electron transport is dominated by the even components of the Green’s function—$\Im \textbf{G}_{ii}$ in the case of Kubo, and $\Re \textbf{G}_{\ell r}$ for Landauer. Crucially, only the former displays a notable dependence on the scattering rate. Indeed, $\Im \textbf{G}_{ii} \propto \Gamma$. As we shall see in the following, this is the most relevant difference between Kubo and Landauer.

## Numerical results

**Fig. 4:**
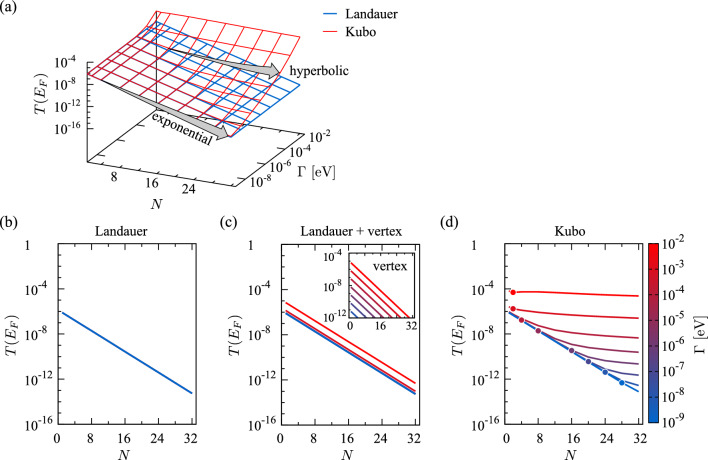
**a** Transmission at the Fermi energy $T(E_F)$ as a function of PA length (unit cells *N*) and electron scattering rate $\Gamma$, within the Landauer and the Kubo formalisms. The exponential scaling $\propto \exp (-\beta N)$ and the $\Gamma$-driven hyperbolic scaling $\propto \Gamma /N^2$ are highlighted. Within Kubo, the exponential scaling disappears for $\Gamma \gtrsim \Gamma_{\cal L} = 2.5 \times 10^{-3}$ eV as, then, transport is dominated by electron–electron scattering processes. Cuts of $T(E_F)$ at fixed $\Gamma$ for the (**b**) Landauer, (**c**) Landauer with vertex corrections, and (**d**) Kubo response. Note that in (**b**) all curves overlap, as Landauer is independent of $\Gamma$. In the inset of (**c**) we show the extra contribution from the vertex corrections, i.e., the difference between the main panels (**c**) and (**b**), which instead depends on $\Gamma$. The filled circles in (**d**) correspond to the crossover length $N_c$ between the exponential and hyperbolic regimes in the Kubo response

**Fig. 5:**
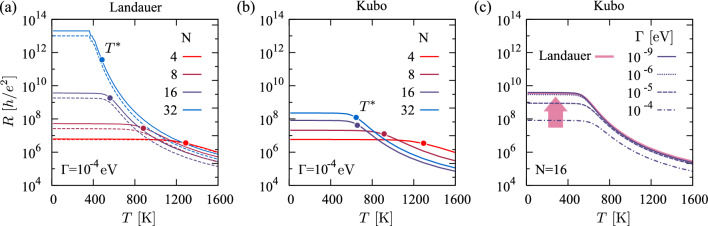
Resistance *R*(*T*) within the (**a**) Landauer without (solid line) and with (dashed line) vertex corrections, and (**b**, **c**) Kubo formalism. **a**, **b** The saturation value below the length-dependent crossover temperature $R(T<T^*)$ is dominated by $T(E_F)$. The filled circles in (**a**, **b**) correspond to the crossover temperature $T^*$, estimated from the resistance inflection point $d^2R(T)/dT^2=0$. **c** For $\Gamma \rightarrow 0$, the Kubo resistance converges towards the Landauer result

We now compare numerical data obtained within the Landauer and the Kubo formalism (see Figs. 4 and  5) and we show that there exist distinct transport regimes, depending on the length of the PA chain *N* (unit cells) and the scattering rate $\Gamma$. Throughout, we set the molecule-to-lead coupling to $\Gamma _\mathcal{L}=\Gamma _\mathcal{R} = 2.5 \times 10^{-3}$ eV, which is representative of a weak chemical bond between PA and metallic (e.g., Au) electrodes through thiol (SH) or amino (NH$_2$) anchoring groups. We first discuss the transmission function, which, generally, displays resonances for poles of the Green’s function, corresponding to the MO of the scattering region. In the following, we will focus on the transmission $T(E_F)$ directly at the Fermi level $E_F$. The transmission, as well as the conductance, $G=e^2/h \times T(E_F)$, defined in terms of the electron charge *e* and Planck’s constant *h*, is mainly controlled by the value of $\Gamma _\mathcal{L}$. While this is evident from the Landauer formula (10), it is less obvious, yet equally true, in the case of the Kubo formula (15), as we show in Sect. 5.1.1.

### Variation with chain length *N*

Within Landauer, we observe an exponential decay in conductance

21 $$\begin{aligned} G = \frac{e^2}{h} \alpha _{\hbox { Landauer}} \exp (-\beta L), \end{aligned}$$

where $L=Nd-a_1$ is the length of the PA chain, the pre-factor $\alpha _{\hbox { Landauer}}$ is representative of the resistance at the molecule-lead interface, and $\beta$ is the attenuation factor. The numerical data are compatible with $\alpha _{\hbox { Landauer}} \sim \Gamma _\mathcal{L}^2$ and $\beta ={0.216}$Å$^{-1}$. The attenuation factor is therewith in fair agreement with the empirical law [81] $\beta = -0.19 + 0.32 \Delta _{\infty }^{1/2} \approx {0.177}$Å$^{-1}$. Further, the Landauer conductance is insensitive to $\Gamma$. Indeed, for $\Gamma \ll \Gamma _\mathcal{L}$, the electron transport is phase-coherent and the Landauer is identical to the Kubo response for short chains, see Fig. 4b and d. For longer chains, scattering processes within the Kubo formalism become dominant and there exists a crossover length scale $L_c = N_c d-a_1$ at which the Kubo response displays a gradual transition between an exponential to a hyperbolic regime, for which we find

22 $$\begin{aligned} G \propto \alpha _{\hbox { Kubo}} L^{-2}, \end{aligned}$$

as expected for non-directional diffusion [82]. The filled circles in Fig. 4d are a guide-to-the-eye to identify the crossover length between the exponential and the hyperbolic decay. The crossover scale $L_c$ decreases as $\Gamma$ increases. The exponential scaling also disappears for $\Gamma \gtrsim \Gamma _\mathcal{L}$ when transport is dominated by scattering at all length scales.

### Temperature dependence

The phenomenological ansatz for finite lifetimes, Eq. (6) essentially describes electron–electron scattering between Landau *quasi*-particles. Within this approximation, the electron Green’s function of the scattering region and, hence, the transmission function, do *not* depend on temperature. Still, there is an effect of temperature on the transport properties, if, instead, we consider the electron current, defined as

23 $$\begin{aligned} I = \frac{e}{h} \int _{-\infty }^{\infty } d\omega \ T(\omega ) \left[ f(\omega -\mu _\mathcal{L})-f(\omega -\mu _\mathcal{R})\right] , \end{aligned}$$

where $f(\omega )$ is the Fermi-Dirac distribution of the lead, which governs thermal activation. The resistance is then obtained as the inverse of the *differential* conductance

24 $$\begin{aligned} R = \left( \frac{dI}{dV_b}\right) ^{-1}. \end{aligned}$$

Within the linear response regime $eV_b \ll \Delta$, it is safe to assume a weak dependence of the transmission function on the external bias, i.e., that $T(\omega ,V_b) \approx T(\omega )$. Moreover, in this regime the current is linear in the external bias, $I \propto V_b$, and the resistance is bias-independent.

In Fig. 5 we show the resistance *R* as a function of temperature for PA chains of different lengths *N*. At high temperatures, we observe an activated behavior corresponding to transitions across the HOMO-LUMO gap $\Delta$. This regime is bounded from below by a characteristic temperature scale, $T^* \propto \Delta /k_B$ that is also controlled by the gap (see the discussion below). The saturation resistance below, R($T<T^*$), is dominated by the transmission at the Fermi energy $T(E_F)$ and thus follows either the exponential (Landauer) or the hyperbolic (Kubo) decay with the chain length. Noteworthy, as $\Gamma \rightarrow 0$, the Kubo resistance converges towards the Landauer result, cf. Fig. 5a and c.

## Discussion

We will now discuss the above numerical results for the finite PA molecules. First, using analytical arguments, we will rationalize the evidenced dependencies on the molecules’ length *N* and scattering rate $\Gamma$. Then, we will put the observed saturation of the molecules’ resistance into relation to recent theories for transport in correlated narrow-gap bulk semiconductors.

### Landauer versus Kubo: analytical insights

The differences between the Landauer and the Kubo transmission function $T(E_F)$ simulated for low energies and temperatures shown in Fig. 4 can be understood from analytical arguments: We symbolically evaluate $T(E_F)$ from Eqs. (7) and (15) and investigate the leading behaviour vis-à-vis the scattering rate $\Gamma$ and the molecule-lead couplings $\Gamma _\mathcal{L}=\Gamma _\mathcal{R}$.

#### The small scattering limit

For a molecular wire with *N* PA unit cells one finds for the Landauer response, Eq. (10), to lowest order in $\Gamma _\mathcal{L}$ and $\Gamma$

25 $$\begin{aligned} T_{\hbox { Landauer}}(E_F;N) = \left( \frac{\Gamma _\mathcal{L}}{t_1}\right) ^2\left( \frac{t_1}{t_2}\right) ^{2N} +\mathcal {O}\left( \Gamma _\mathcal{L}^n\Gamma ^m\right) \end{aligned}$$

with $n\ge 2,\ n+m\ge 4$. For the Kubo response, Eq. (15), the leading order comes out as exactly the same,

26 $$\begin{aligned} T_{\hbox { Kubo}}(E_F;N)= \left( \frac{\Gamma _\mathcal{L}}{t_1}\right) ^2\left( \frac{t_1}{t_2}\right) ^{2N}+\mathcal {O}\left( \Gamma _\mathcal{L}^p\Gamma ^q\right) \end{aligned}$$

albeit with $p\ge 0$, $p+q\ge 2$. Terms beyond the leading order will be discussed in Sect. 5.1.2.

The above expansions readily explain why the simulated Landauer and Kubo transmissions (Fig. 4) coincide in the limit of vanishing $\Gamma$, irrespective of the chain length *N*. In fact, in both cases, the lowest order term in the transmission is proportional to $\Gamma _\mathcal{L}^2$ and *independent* of $\Gamma$. For the typical case $\Gamma _\mathcal{L}\gg \Gamma$, the above leading term is a good approximation (lower bound) for the transmission $T(E_F)$ in the zero temperature limit, where the scattering rate $\Gamma$ is expected to become small. Crucially, a finite coupling $\Gamma _\mathcal{L}$ leads to a residual transmission and, hence, a finite conductance $G(T\rightarrow 0)>0$, which will be discussed in Sect. 5.2 below.

#### Sizable scattering $\Gamma$

Above a crossover value $N_c$, we observe a strong deviation between the Landauer and the Kubo transmission, with the Kubo one decaying much more slowly for $N\rightarrow \infty$. The $\Gamma$-dependence of this phenomenon (see Fig. 4a, c) provides a definite clue as to its microscopic origin. Indeed, while the leading order terms of Landauer and Kubo (discussed above) coincide, terms beyond the low-scattering limit are different, see the different powers in the corrections indicated in Eqs. (25), (26).

By construction, the lowest order in scattering within Landauer is $\Gamma _\mathcal{L}^2$, i.e., there are no pure-$\Gamma$ terms and no mixed terms $\propto \Gamma _\mathcal{L}\Gamma$. Within Kubo, on the other hand, there are both pure-$\Gamma$ terms (the lowest order is $\Gamma ^2$) and a mixed term $\Gamma _\mathcal{L}\Gamma$. Plausibly assuming $\Gamma _\mathcal{L}\gg \Gamma$, this mixed term will dominate the corrections to the leading $\propto (\Gamma _\mathcal{L})^2$ term in the Kubo transmission Eq. (26). Expanding to this next order, we find

27 $$\begin{aligned} T_{\hbox { Kubo}}(E_F; N)= c_1(N) \Gamma _\mathcal{L}^2 + c_2(N) \Gamma _\mathcal{L}\times \Gamma +... \end{aligned}$$

with, for even *N*,

28 $$\begin{aligned} c_1(N)&=\frac{1}{N^2 t_1^2}\left( \frac{t_1}{t_2} \right) ^{2N}\end{aligned}$$

29 $$\begin{aligned} c_2(N)&=4\times \frac{\sum _{n=1}^N \left[ (n-1)a_1+na_2\right] ^2 t_1^{2(n-1)}t_2^{2(N-n)}}{ \left[(N-1)a_1+Na_2\right]^2 t_2^{2N}} \end{aligned}$$

Since $t_2>t_1$, the coefficient $c_1(N\rightarrow \infty )\propto (t_1/t_2)^{2N}$ is suppressed exponentially, while

30 $$\begin{aligned} \lim _{N\rightarrow \infty } c_2(N)=4\times \frac{a_2^2t_2^4+(a_1^2+4a_1a_2+a_2^2)t_2^2t_1^2+a_1^2t_1^4}{(a_1+a_2)^2(t_2^2-t_1^2)^3} \times \lim _{N\rightarrow \infty }\frac{1}{N^{2}} >0 \end{aligned}$$

merely decays hyperbolically. For small $\Gamma$ and *N*, the transmission is dominated by $c_1(N)\Gamma ^2_\mathcal {L}$ leading to $T_{\hbox { Kubo}}\approx T_{\hbox { Landauer}}$. Owing to the different scaling with system size, however, the $c_2(N)\Gamma _{\mathcal {L}}\Gamma$ term (absent in Landauer) is gaining importance for longer molecules, all the more rapid the larger $\Gamma$. Indeed, for a given $\Gamma$, there must be a crossover length $N_cd$, above which the $c_2(N)\Gamma _{\mathcal {L}}\Gamma$ term surpasses $c_1(N)\Gamma ^2_\mathcal {L}$. For $N>N_c$ the $\Gamma _\mathcal{L}\Gamma$ term then dominates the transmission, turning the molecule’s scattering rate $\Gamma$ into a relevant energy scale and causing a strong deviation from Landauer’s transmission.

While the $\Gamma _{\mathcal {L}}\Gamma$ term is absent in the plain Landauer formalism, the vertex corrections to the lead-molecule coupling introduce such a term. However, as is evident from Eq. (14) the vertex corrections only renormalize the pre-factor, not the structure and length-dependence of the tunneling processes. Therefore, the vertex-corrected Landauer transmission still decays exponentially with system size, see Fig. 4.

The reason for the very slow decay of the *Kubo* conductance with the chain length is the incoherence (finite lifetime) of charge carriers inside the molecule. Indeed, as discussed in Sect. 3.3, the ingredients entering the transmission function are significantly more $\Gamma$-dependent within the Kubo formalism than within Landauer’s. Intuitively, the finite lifetimes of the HOMO and LUMO states lead to an energy broadening, which smoothes the gap edges and spills spectral weight into the gap [83, 84]. These incoherent carriers can then diffuse through the molecule, which is described by the sequential hopping processes that make up the Kubo response (visualized in Fig. 2). In this sense, the length scale $L_c=N_cd-a_1$ (see Sect. 4.1) separates regimes in which molecular transport dominantly occurs through tunneling ($L\lesssim L_c$) and diffusion ($L\gtrsim L_c$), respectively.

### Resistance saturation in semi-conducting molecules

At elevated temperatures, the resistances in Fig. 5 exhibit, as expected, an Arrhenius regime, in which conduction through the semi-conducting molecular chain is activated. The largest resistance is then obtained for the shortest molecules, owing to their larger gaps. Cooling below a crossover temperature $T^*$, however, the resistance from, both, Landauer and Kubo cedes to increase exponentially and, instead, levels off to saturate towards $T\rightarrow 0$. This saturation phenomenon has first been discussed [23] for correlated narrow-gap semiconductors [25], such as Kondo insulators. In these periodic bulk systems, the low-temperature regime is dominated by *intra-band* transitions [24]: For a band $\epsilon _{\textbf{k}}^0$ endowed with a quasi-particle lifetime $\hbar /(2\Gamma )$ and weight *Z*, the Kubo conductivity (without vertex corrections) can be expressed analytically as [85]

31 $$\begin{aligned} \sigma (T)=\frac{e^2\hbar }{2\pi ^2V}\frac{Z^2}{\Gamma }\frac{1}{k_BT} \sum _{\textbf{k}}(v_{\textbf{k},x})^2 \left( \Re \Psi ^\prime (z) -\frac{\Gamma }{2\pi k_BT}\Re \Psi ^{\prime \prime }(z) \right) \end{aligned}$$

with the unit cell volume *V*, the intra-band group velocity

32 $$\begin{aligned} v_{\textbf{k},x}=1/\hbar \nabla _{k_x}\epsilon _{\textbf{k}}^0 \end{aligned}$$

with $\textbf{k}$ in the Brillouin zone and derivatives of the digamma function $\Psi (z)$ evaluated at $z=1/2+(\Gamma +\imath \epsilon _{\textbf{k}})/(2\pi k_BT)$, where $\epsilon _{\textbf{k}}=Z\epsilon _{\textbf{k}}^0$. The characteristic temperature $T^*$ that delimits the resistivity saturation regime encoded in the Kubo Eq. (31) can be crudely estimated as [23]

33 $$\begin{aligned} \hbox {intra-band:}\qquad k_BT^*=\frac{1}{\sqrt{10}\pi }\left( \frac{\Delta }{2}+\frac{11}{5}\frac{\Gamma ^2}{\Delta }+\mathcal {O}(\Gamma ^4)\right) \end{aligned}$$

where $\Delta$ is the renormalized charge gap.^3^ Note that the crossover from an activated behaviour to a low-temperature conductance regime with weak temperature dependence was recently also suggested based on the Meir-Wingreen formula applied to a single molecular level [86].

The digamma function accounts for thermal ($k_BT$) and lifetime ($\tau =\hbar /(2\Gamma )$) broadening on an equal footing. For infinitely long-lived charge carriers, $\Gamma \rightarrow 0$, Eq. (31) reduces—to leading $1/\Gamma$ order—to the Boltzmann conductivity, since [85]

34 $$\begin{aligned} \frac{1}{2\pi ^2k_BT} \Re \Psi ^\prime \left( \frac{1}{2}+\frac{\imath \epsilon _{\textbf{k}}}{2\pi k_BT}\right) = f^\prime (\epsilon _{\textbf{k}}). \end{aligned}$$

In that semi-classical limit, the scattering rate $\Gamma$ becomes a mere prefactor and the response is activated for all temperatures. In that sense, the resistance saturation found, here, in molecules within both the Kubo and the Landauer formalism is a quantum effect.

The above formulas in Eqs. (31, 32) describe charge transport from transitions taking place within the same band $\epsilon _{\textbf{k}}$ that disperses owing to unit cell-to-unit cell hopping. Already for periodic lattice models with multiple atoms per unit cell, one has to extend this setting, allowing in particular for inter-band and intra-unit cell transitions. For the Fermi velocities, this is achieved in the generalized Peierls approach [79], in which

35 $$\begin{aligned} (\textbf{v}_{\textbf{k},x})_{nm}=\frac{1}{\hbar }\bigl [\underbrace{\nabla _{k_x}(\textbf{H}_{\textbf{k}})_{nm}}_{\mathrm {inter-unit\ cell}} - \underbrace{\imath \left( x_n-x_m \right) (\textbf{H}_{\textbf{k}})_{nm}}_{\mathrm {intra-unit\ cell}}\bigr ] \end{aligned}$$

where $(\textbf{H}_{\textbf{k}})_{nm}$ is the Hamiltonian expressed in a local basis, with $n=(i,l)$ indexing, both, the hosting atomic site *i* and orbital *l*.^4^ The above formula has the virtue of “interpolating" between the momentum-space description of a periodic system (first term: inter-unit cell transitions) and the large real-space unit cells with open boundary conditions of finite molecules (second term: intra-unit cell transitions). Indeed, Eq. (35) assures that transport observables for a periodic solid with a primitive one-atomic unit cell can be equivalently described by a non-primitive unit cell that has been, say, doubled in the *x* direction.

In finite systems (i.e., with open boundary conditions), such as the SSH model Eq. (1), the real-space formulation manifestly only involves inter-orbital inter-atomic transitions and the Fermi velocity reduces to the second term, and with $({\textbf{H}}_{\textbf{k}})_{nm} = -t_{nm}$ for ${\textbf{k}}=(0, 0, 0)$, to Eq. (16). Likewise, Eq. (31) has to be replaced with the (lengthy) expression for inter-band transitions, which can be found in Eqs. (3, 11, 29) of Ref. [37]. Using the same procedure as above, we can crudely estimate the dependencies of the resistance saturation regime, finding

36 $$\begin{aligned} \hbox {inter-band:}\qquad k_BT^*=\frac{1}{\sqrt{2}\pi }\left( \frac{\Delta }{2}+3\frac{\Gamma ^2}{\Delta }+\mathcal {O}(\Gamma ^4)\right) . \end{aligned}$$

A comparison to Eq. (33) reveals that the saturation regime, $T<T^*$, is roughly larger by a factor of two for gapped extended systems than in periodic semiconductors with the same gap $\Delta$. According to Eq. (36), the dominant control parameter for $T^*$ is the charge gap $\Delta$, explaining why shorter chains (with their larger $\Delta$, see Fig. 1c) exhibit a basically flat resistance up to far beyond room temperature. The scattering rate $\Gamma$ only has a sub-leading effect on $T^*$, in congruence with the numerical data in Fig. 5c. From the arguments presented in Sect. 5.1.2 for the transmission function we further understand, that the Kubo resistance converges towards the Landauer result in the limit $\Gamma \rightarrow 0$.

## Conclusion

Using realistic parameters to model semi-conducting polyacetylene molecular wires, our results suggest that a resistance saturation regime, extending up to at least room-temperature, should be commonly observed—provided that extrinsic factors (disorder, doping) do not destroy the charge gap. The residual conduction is provided by incoherent spectral weight spilling into the HOMO-LUMO gap. We find that the extent of the temperature regime, $0\le T\le T^*$, that exhibits saturation is directly controlled by the size of the gap $\Delta$. Thus, for a given temperature, recovering a more conventional activated (Arrhenius) type of conduction should be possible by straining the molecular wire [86, 89]. This strain tuning of the crossover temperature $T^*$ separating both regimes is analogous to the pressure tuning of transport in, e.g., Kondo insulators [23, 90, 91]. The only conceptual difference between resistivity saturation in these 3D bulk systems and resistance saturation in the extended 1D wire is conduction being dominated, respectively, by intra- and inter-band transitions that live on different energy scales, namely $\sim \Delta /2$ and $\sim \Delta$. Thus, for a given gap, the resistance plateau in extended systems is much wider in temperature than for periodic systems. We further stress that the resistance plateau is sizable even for extremely small scattering rates. It is thus not inconceivable that already the inclusion of electron–phonon scattering from zero-point motion could be sufficient to create it.

While results from the Landauer approach and Kubo linear response theory oftentimes appear to be similar, we find qualitative differences to abound when the scattering rate is non-negligible and the molecule is long. In particular, the Landauer (Kubo) conductance decays exponentially (hyperbolically) with system size. At low temperatures, the Kubo response is far more sensitive to the charge carriers’ incoherence than the Landauer approach. For a given scattering rate, even if the crossover temperature $T^*$ is comparable, the saturation value of the Kubo resistance can be orders of magnitude smaller—especially for long molecules. Experimentally observing the length-induced crossover from ballistic to a diffusive transmission in semiconducting polyacetylene wires might be challenging, because the effect of electronic scattering manifests only for chains of considerable length.

On the level of methodology, a clear hierarchy of transport approaches for carriers with finite lifetimes emerges from our discussion. In order of decreasing lifetimes $\tau =\hbar /(2\Gamma )$, the methodology required to fully capture signatures of electronic incoherence escalates as follows: Boltzmann $\rightarrow$ Landauer $\rightarrow$ Kubo. Indeed, for a diminishing scattering rate $\Gamma$, results from Kubo converge to those of Landauer. For infinitely long-lived quasi-particles, the coefficient of the leading $1/\Gamma$ order reduces to the Boltzmann expression. In all, our model calculations provide guidance for the choice of transport methodology in future *ab initio* simulations for semi-conducting molecular systems.

## Appendix A The macroscopic limit of the linear undimerized chain ($t_1=t_2$)

We find it instructive to derive relevant elements of the Green’s function for the linear undimerized chain that is obtained from the SSH model for $t_1=t_2\equiv t$ in the limit of long chains. Note that the latter (in contrast to the SSH model) will be metallic. For an undimerized chain of *N* sites the Hamiltonian has the form of a symmetric tridiagonal Toeplitz matrix

A.1 $$\begin{aligned} {\textbf{H}}^{[N]}= \begin{pmatrix} 0 &{} -t &{} 0 &{} \cdots \\ -t &{} 0 &{} -t &{} \\ 0 &{} -t &{} 0 &{} \ddots \\ \vdots &{} &{} \ddots &{} \ddots \end{pmatrix} \in \mathbb {R}^{N\times N}. \end{aligned}$$

In matrix notation the corresponding Green’s function then is $({\textbf{G}}^{[N]})^{-1}(z)=z{\textbf{I}}^{[N]}-{\textbf{H}}^{[N]}$. While the general inversion of $({\textbf{G}}^{[N]})^{-1}$ in terms of Chebyshev polynomials of the second kind is well-known [92, 93], we will here follow two different paths.

In summary, the results are: In the macroscopic limit, $N\rightarrow \infty$, the density of states in the middle of the molecule, unsurprisingly, converges to that of the gapless periodic 1D bulk system. The DOS at the extremities of the very long but finite and non-periodic one-dimensional chain becomes semi-circular. Intriguingly, the same DOS is obtained for the infinite dimensional Bethe lattice [45, 94].

### A.1 The ends of the long linear chain

For Green’s function elements at (or connecting) the borders, we follow arguments from Schur’s complement. If

A.2 $$\begin{aligned} {\textbf{F}}= \begin{pmatrix} {\textbf{F}}_{11} &{} {\textbf{F}}_{12} \\ {\textbf{F}}_{21} &{} {\textbf{F}}_{22} \end{pmatrix} \end{aligned}$$

with matrices ${\textbf{F}}_{11}$ and ${\textbf{F}}_{22}$ that are of square shape, and, further, ${\textbf{F}}_{11}$ and ${\textbf{B}}={\textbf{F}}_{22}-{\textbf{F}}_{21}{\textbf{F}}_{11}^{-1}{\textbf{F}}_{12}$ that are invertible, one can show that

A.3 $$\begin{aligned} {\textbf{F}}^{-1}= \begin{pmatrix} {\textbf{F}}_{11}^{-1}+{\textbf{F}}_{11}^{-1}{\textbf{F}}_{12}{\textbf{B}}^{-1}{\textbf{F}}_{21}{\textbf{F}}_{11}^{-1} &{} -{\textbf{F}}_{11}^{-1}{\textbf{F}}_{12}{\textbf{B}}^{-1} \\ -{\textbf{B}}^{-1}{\textbf{F}}_{21}{\textbf{F}}_{11}^{-1} &{} {\textbf{B}}^{-1} \end{pmatrix} \end{aligned}$$

Setting up ${\textbf{F}}^{[N+1]}=({\textbf{G}}^{[N+1]})^{-1}$, the inverse Green’s function for $N+1$ sites, from ${\textbf{F}}_{11}=z{\textbf{I}}^{[N]}-{\textbf{H}}^{[N]}={\textbf{F}}^{[N]}\in \mathbb {C}^{N\times N}$, ${\textbf{F}}_{22}=z$, and ${\textbf{F}}_{21}=(0,0,\cdots ,0,-t)\in \mathbb {R}^{1\times N}$, with ${\textbf{F}}_{12}=({\textbf{F}}_{21})^T\in \mathbb {R}^{N\times 1}$, one easily finds the recursion relations

A.4 $$\begin{aligned} ({\textbf{G}}^{[N+1]})_{N+1,N+1} = \frac{1}{z-t^2 ({\textbf{G}}^{[N]})_{N,N}} \end{aligned}$$

and

A.5 $$\begin{aligned} ({\textbf{G}}^{[N+1]})_{1,N+1} = -\frac{t({\textbf{G}}^{[N]})_{1,N}}{z-t^2({\textbf{G}}^{[N]})_{N,N}} \overset{N\ge 2}{=}\ ({\textbf{G}}^{[2]})_{1,2} \prod _{M=2}^{N+1}(-t) ({\textbf{G}}^{[M]})_{M,M} \end{aligned}$$

For the diagonal component, Eq. (A.4), one easily solves the recursion in the $N\rightarrow \infty$ limit and, interestingly, finds

A.6 $$\begin{aligned} \left( \textbf{G}^{[\infty ]}(z)\right) _{\ell \ell /rr}=\lim _{N\rightarrow \infty }({\textbf{G}}^{[N]})_{N,N}=\frac{z}{2t^2}-\frac{1}{2t^2}\sqrt{z^2-4t^2} \end{aligned}$$

for the infinite chain with open boundary conditions. With $z=\omega +i\eta$, to obtain the non-interacting retarded Green’s function, one then sees that the DOS at the borders of the infinitely-long *one-dimensional* chain

A.7 $$\begin{aligned} -\frac{1}{\pi }\left( \Im \textbf{G}^{[\infty ]}(\omega )\right) _{\ell \ell /rr} =\frac{1}{2\pi t^2}\sqrt{4t^2-\omega ^2} \end{aligned}$$

is semi-circular, as is also the case for the *infinite dimensional* Bethe lattice [45, 94].

### A.2 The centre of the long linear chain

For information on the centre of the linear molecule, we devise a different, physics-oriented, strategy: Using a chain with an odd number of atoms, $N=2m+1$ with $m\in \mathbb {N}$, we consecutively integrate out the outermost sites to obtain an effective Green’s function for the center three sites:

A.8 $$\begin{aligned} \left({\textbf{G}}^{[N]}\right)^{-1}= \begin{pmatrix} z &{} t &{} 0 &{} \cdots &{} &{} \cdots &{} 0\\ t &{} \ddots &{} \ddots &{} \ddots &{} &{} &{} \vdots \\ 0 &{} \ddots &{} {z} &{} t &{} 0\\ \vdots &{} \ddots &{} {t} &{} z &{} t &{} \ddots &{} \vdots \\ &{} &{} {0} &{} {t} &{} z &{} \ddots &{} 0\\ \vdots &{} &{} &{} \ddots &{} \ddots &{} \ddots &{} t \\ 0 &{} \cdots &{} &{} \cdots &{} 0 &{} t &{} z \end{pmatrix}_{N\times N} \!\!\!\!\!\!\!\!\!\!\!\! \longrightarrow \left({\textbf{G}}_{{\hbox {eff}}}^{[N]}\right)^{-1}= \begin{pmatrix} z_N &{} t &{} 0 \\ t &{} z &{} t \\ 0 &{} t &{} z_N \end{pmatrix} \end{aligned}$$

Using the path-integral formalism, it is quite elementary to show, for $N\ge 5$, the recursion formula $z_N=z-t^2/z_{N-2}$. In the limit of large *N*, the solution is

A.9 $$\begin{aligned} z_{N\rightarrow \infty }=z/2\pm \sqrt{z^2/4-t^2} \ \ \textrm{for} \ \ \Re z\gtrless 0. \end{aligned}$$

Then, for the centre of the molecule

A.10 $$\begin{aligned} \left(\textbf{G}^{[N]}\right)_{\hbox {centre}}=\left(\textbf{G}_{\hbox {eff}}^{[N]}\right)_{2,2}=\frac{1}{z-2t^2/z_N}\overset{N\rightarrow \infty }{\longrightarrow }\frac{1}{\sqrt{z^2-4t^2}}, \end{aligned}$$

whence

A.11 $$\begin{aligned} -\frac{1}{\pi } \left( \Im \textbf{G}^{[\infty ]}(\omega )\right) _{\hbox {centre}}=\frac{1}{\pi }\frac{1}{\sqrt{4t^2-\omega ^2}}, \end{aligned}$$

which is the same as the local DOS of the dispersion, $\epsilon (k)=-2t\cos (k a)$, of a periodic one-dimensional system with nearest-neighbour hopping *t*.

These results for the (metallic) undimerized chain could be generalized to the (gapped) dimerized chain. Basically, there will be an alternation of hoppings in the continued fraction recursions, that will lead to the gapping and results very close to the numerical data shown for $N=32$ in Fig. 1f.

## Footnotes

1 Within a tight-binding (or Hückel) framework, with an orthogonal basis and short-range hopping, this condition is usually verified, whereas, within more sophisticated frameworks, e.g., density functional theory, this requires the scattering region to be sufficiently extended, typically including also a few layers of the electrodes.

2 Because of the chosen termination of the wire, the molecule consists of *N* unit cells, *minus* one single bond of length $a_1$, as stressed previously by Nikolić [77].

3 One approximates the dispersion as $\epsilon _{\textbf{k}}=\Delta /2$, expands $\sigma (T)\approx \sigma _{\hbox { low-T}}(T)=\sigma _0+\sigma _2T^2$ to second order, and solves for the inflection point $T^*$ in $R(T)=1/\sigma _{\hbox { low-T}}(T)$. This procedure overestimates $T^*$ but gets right the trends with $\Gamma$ and $\Delta$.

4 The Peierls approach yields observables that are basis-dependent [87]. It has been shown [88] that the most accurate description is obtained in a maximally localized basis.

## Declarations

### Data availability

The datasets generated and analysed during the current study are available from the authors upon reasonable request.
